# Impacts of Herbal Medicine Use on Lipid Profiles in Type 2 Diabetic Patients in Northwest Ethiopia: A Comparative Cross‐Sectional Study

**DOI:** 10.1155/bmri/1020727

**Published:** 2026-01-21

**Authors:** Assefa Belay Asrie, Tafere Mulaw Belete, Tezera Jemere Aragaw, Melshew Fenta Misker, Alemante Tafese Beyna, Habtamu Semagne Ayele, Kidist Goshime Tekle, Yonas Zewdu Milikit, Ephrem Adane Andargie, Hiwot Tesfaselassie Afework, Yenatfanta Gezu Lenjiso, Gebrehiwot Lema Legese

**Affiliations:** ^1^ Department of Pharmacology, School of Pharmacy, College of Medicine and Health Sciences, University of Gondar, Gondar, Ethiopia, uog.edu.et; ^2^ Department of Pediatrics and Child Health, School of Medicine, College of Medicine and Health Sciences University of Gondar, Gondar, Ethiopia; ^3^ Department of Ophthalmology, School of Medicine, College of Medicine and Health Sciences, University of Gondar, Gondar, Ethiopia, uog.edu.et; ^4^ Department of Gynecology and Obstetrics, School of Medicine, College of Medicine and Health Sciences, University of Gondar, Gondar, Ethiopia, uog.edu.et; ^5^ Department of Anesthesiology, Critical Care and Pain Medicine, School of Medicine, College of Medicine and Health Sciences, University of Gondar, Gondar, Ethiopia, uog.edu.et; ^6^ Department of Internal Medicine, School of Medicine, College of Medicine and Health Sciences, University of Gondar, Gondar, Ethiopia, uog.edu.et

**Keywords:** herbal medicine, herbal medicine use, lipid profiles, lipid ratios, medicinal plants, Type 2 diabetes mellitus

## Abstract

**Background:**

Blood lipid abnormalities are common among Type 2 diabetes mellitus (T2DM) patients, and achieving better glycemic control may help improve their lipid profiles. Concomitant use of herbal medicines with conventional antidiabetic medications is a common practice among T2DM patients in Ethiopia. This study was conducted to evaluate the impacts of herbal medicine use on lipid profiles among T2DM patients.

**Method:**

This is a cross‐sectional study and was conducted from May 01 to July 30, 2024. A sample of 416 participants was approached for the study. The sample size was calculated using a single population proportion formula. A systematic random sampling method was used to select the participants. The data were collected through interviewer‐administered questionnaire and patient medical record reviews. Patients were randomly selected, and their corresponding medical records, retrieved from the archive based on follow‐up schedules of the patients, were accessed and reviewed using a data collection tool adapted from previous studies. Lipid parameters, including total cholesterol (TC), triglycerides (TG), low‐density lipoprotein cholesterol (LDL‐C), and high‐density lipoprotein cholesterol (HDL‐C) levels, as well as TC/LDL‐C, TG/LDL‐C, and LDL‐C/HDL‐C ratios and atherogenic index of plasma (AIP), were compared between herbal medicine users and nonusers using the Mann–Whitney *U* Test and linear regression analysis.

**Results:**

Of the participants approached, 381 (91.6%) were included in the study. Among the participants included in the study, 141 (37.0%) reported having used herbal medicine since they were diagnosed with diabetes, and almost all were active users at the time of the study. The median TC, TG, and LDL‐C levels and TC/HDL‐C, TG/HDL‐C, and LDL‐C/HDL‐C ratios and AIP of herbal medicine users were significantly lower than those of nonusers (*p* < 0.01), whereas the median HDL‐C was significantly higher (*p* < 0.05). Moreover, linear regression analyses indicated that the TC, TG, and LDL‐C levels were decreased by 6.84 mg/dL (*β* = −6.84, *p* < 0.05), 8.69 mg/dL (*β* = −8.69, *p* < 0.01), and 6.75 mg/dL (*β* = −6.75, *p* < 0.05), respectively, whereas HDL‐C values increased by 1.59 (*β* = 1.59, *p* < 0.05) in herbal drug users as compared with nonusers. Similarly, compared with nonusers, TC/HDL‐C, TG/HDL‐C, and LDL‐C/HDL‐C ratios in herbal medicine users were reduced by 0.32 (*β* = −0.32, *p* < 0.01), 0.34 (*β* = −0.34, *p* < 0.01), and 0.23 (*β* = −0.23, *p* < 0.05), respectively, whereas AIP decreased by 0.041.

**Conclusion:**

In conclusion, the use of herbal medicines was associated with significant reductions in TC, TG, and LDL‐C levels, as well as in the TC/HDL‐C, TG/HDL‐C, and LDL‐C/HDL‐C ratios and AIP, whereas also associated with a significant increase in HDL‐C levels. The results imply that herbal remedies may have beneficial effects in optimizing serum lipid levels in T2DM patients and could ultimately help reduce associated cardiovascular risks. However, because this study was cross‐sectional and carried out at a single site, we recommend conducting more rigorous, multicenter observational and trial studies to generate more comprehensive and conclusive results.

## 1. Background

Diabetes mellitus is characterized by hyperglycemia, which in turn serves as the basis for other metabolic abnormalities [1, 2]. Abnormalities in blood lipid levels are common among patients with Type 2 diabetes mellitus (T2DM), with a prevalence of 72%–85% [3, 4]. Elevated triglycerides (TG) and lowered high‐density lipoprotein (HDL‐C) are the primary lipid abnormalities in diabetic‐related dyslipidemia [5]. These lipid abnormalities play a crucial role in the formation of arterial plaque, known as atherosclerosis [6, 7]. Consequently, diabetic patients have an increased probability of coronary artery disease compared with diabetes‐free individuals [3]. Moreover, dyslipidemia in diabetic patients may advance to more severe problems, including micro‐ and macrovascular complications. These complications significantly compromise the quality of life of the patients and may eventually lead to life‐threatening conditions such as stroke [8, 9].

Lifestyle changes, including dietary modifications and aerobic exercise, are the initial approaches to diabetes dyslipidemia management [10]. Attaining good glycemic control using diet and antidiabetic medications can also improve lipid parameters [11–13]. Lipid‐lowering drugs should be used when the condition remains uncontrolled by these approaches [14]. In this regard, statins are considered first‐line treatments. However, some patients do not achieve the target LDL‐C level and need combination therapy with other lipid‐lowering agents such as ezetimibe, fibrates, and icosapent ethyl [15], and even with sufficient LDL‐C reduction through statin therapy, many patients remain at high risk of CVD [16]. Moreover, although several lipid‐lowering drugs have been approved for clinical use, they may have efficacy problems and be associated with a variety of side effects, including hepatotoxicity, rhabdomyolysis, skin reactions, and gastrointestinal disturbances [17–19]. Herbal medicines are used as alternative and complementary agents for the management of dyslipidemia because of minimal toxicities and useful biological effects, such as antioxidant and anti‐inflammatory properties [20]. They act on various targets and have multiple pharmacological effects because they contain a variety of phytochemicals [21]. Herbal remedies are, therefore, expected to work in concert to optimize lipid levels [20]. Herbal medicines intended for the treatment of diabetes may impact the lipid profiles secondary to their antihyperglycemic effect or direct antihyperlipidemic mechanisms and antioxidant activities, which are beneficial in lipid abnormalities [22–24]. Evidence on the useful biological effects of medicinal plants is mainly based on experimental or clinical trial studies under controlled circumstances. Therefore, it is imperative to assess the clinical outcomes of herbal drug use, such as lipid profiles among diabetic patients practicing herbal medicine use under normal and uncontrolled circumstances as part of their diabetic care.

Previous studies conducted in Ethiopia demonstrated that concomitant herbal medicine use with conventional medications is common among patients with T2DM [25–28]. Many medicinal plants have been used for the treatment of diabetes in the traditional medical practice of Ethiopia, and many of them were studied for their antidiabetic activities and phytochemical contents [29–31]. According to existing literature, herbal medicines may have beneficial effects on lipid profiles among diabetic patients due to their antihyperglycemic or other biological activities [12, 13, 20, 32–34]. However, from literature searches, evidence about effects on clinical outcomes, including lipid profiles, is lacking in Ethiopian health settings. This was the foundation for planning and conducting this study. Thus, this study is aimed at assessing the effects of herbal medicine use on lipid profiles among patients with T2DM under uncontrolled real practical conditions in which they incorporated the treatments into their diabetic care.

The study is expected to have considerable significance to investigators, other researchers, the study population, and the general public. For the investigators, the study serves as an opportunity to enhance skills in clinical research, data analysis, and multidisciplinary teamwork, particularly in the context of traditional medicine use and its clinical implications among patients with diabetes and other chronic conditions. Besides, the study may give important baseline data on the impacts of herbal medicine use on lipid profiles in patients with T2DM. The comparative results could be used as baseline data by the investigators and other researchers for future studies, including longitudinal studies and clinical trials, and may also contribute to evidence‐based integrated patient care. For the study population and the public, the findings may provide insights into the potential health benefits of complementary herbal medicine use under real‐world conditions. This could promote evidence‐based use of herbal medicines and support strategies that integrate traditional health practices into the conventional healthcare system, ultimately improving health outcomes in diabetic care.

## 2. Methods

### 2.1. Study Setting and Period

The study was carried out at the University of Gondar Comprehensive Specialized Hospital (UoGCSH), which is located in Amhara National Regional State, Northwest Ethiopia, 738 km from Addis Ababa, the country′s capital. It is the only referral, educational, and research facility in the Central Gondar Zone of Northwest Ethiopia. It provides healthcare services for more than 13 million people in Central Gondar and neighboring zones. The study participants were recruited at the hospital′s chronic illness clinic, one of its several outpatient clinics, from May 01 to July 30, 2024. The clinic offers services to patients with a range of chronic conditions, including diabetes [35].

### 2.2. Study Design

A hospital‐based cross‐sectional study design was used to evaluate the impacts of herbal medicine use on lipid profiles among T2DM patients, compared with those who do not use herbal medicines.

### 2.3. Source and Study Population

T2DM patients on follow‐up care at the chronic illness clinic in the hospital were the source population. The study population was the subset of this population selected according to the sampling procedure and eligibility criteria and ultimately included in the study.

### 2.4. Eligibility Criteria

Being 18 years of age or older and having at least two previous follow‐up visits at the hospital were considered as initial recruitment criteria to collect data through interviews. Then, to be eligible for inclusion in the final analysis, they needed to have fasting blood glucose and lipid profile results recorded consecutively during their last three visits in their medical records (two previous visits and during the data collection time). Patients with missing outcome data were excluded from the final analysis. Having a diagnosed psychiatric disorder or any other mental disorder that limits the participants′ communication and unwillingness to participate were the exclusion criteria for the study.

### 2.5. Sample Size Determination and Sampling Technique

The sample size of the study participants (*N*) was determined by using a single population proportion formula considering the following assumptions: prevalence (*p*) of herbal medicine use 62%, standard normal distribution value at a 95% confidence level of Z/2 = 1.96, and a margin of error (*w* = 5*%*). This gives a sample size of 362 patients, as shown below.

N=Z2p1−pw2=1.9620.6210.62−0.052=362.03362≈ patients.

A previous study conducted at our study site reported that 62% of T2DM patients used herbal medicines [25], and this proportion was used to calculate the sample size. The prevalence from this specific study was selected because it was conducted at the same study site, ensuring alignment with local prevalence. Once the minimum sample size was calculated, a 15% contingency was added for the nonresponse rate, and the total sample size was determined to be 416.

According to the record (logbook) of the follow‐up clinic, on average, 20 T2DM patients visit the clinic every working day for clinical checkups and medication refills. Based on the sample size of the study, the number of patients to be interviewed and their charts reviewed per day during the working days of the 3‐month data collection period was estimated to be six. The number of patients who visit the clinic every working day was divided by the number of participants to be interviewed per day. Based on this, every third of the T2DM patients attending the follow‐up clinic during the data collection period was selected randomly based on their appearance at the follow‐up clinic. The 3‐month period for the data collection was used to give the chance of selection to possibly all of the patients, as almost all of the patients return to the clinic within the 3‐month period. We also carefully managed to avoid reconsideration of a single patient.

### 2.6. Variables

#### 2.6.1. Independent Variable

Herbal medicine use practice was the independent variable, and its impacts on lipid profiles were assessed by comparing users with nonusers.

#### 2.6.2. Confounding Variables

Sex, age, residence, marital status, educational status, occupation, disease duration, dietary recommendations, physical exercise, health insurance coverage, alcohol use, comorbid conditions, diabetic complications, fasting blood glucose level, antidiabetic drug regimen, and statin drug use were the confounding variables. Smoking and body mass index (BMI) were not included as potential confounders; smoking was reported by only 4.7% of the participants, and BMI data were unavailable for nearly all participants.

#### 2.6.3. Dependent Variables

Total cholesterol (TC), triglycerides (TG), low‐density lipoprotein cholesterol (LDL‐C), high‐density lipoprotein cholesterol (HDL‐C) levels, and lipid ratios were the dependent outcome variables. These lipid levels represent the averages of laboratory test results from three follow‐up visits, as recorded in the patients′ medical files. The lipid ratios are the ratios of TC, TG, or LDL‐C levels to HDL‐C values. The atherogenic index of plasma (AIP) was also included as a dependent variable and calculated as follows [36].

Atherogenic index of plasma AIP=log10Triglycerides mg/dLhigh−density lipoprotein cholestrol mg/dL.

### 2.7. Analyzing Lipid Profiles

Lipid profile data were obtained from clinical records. The analyses/measurements were performed by the hospital′s clinical chemistry laboratory as part of the patients′ routine follow‐up care. Lipid serum concentrations, including TC, TG, LDL‐C, and HDL‐C, were analyzed using a Beckman Coulter automated chemistry analyzer, following the standard operating procedures of the manufacturer. For this study, three consecutive lipid profile test results, two previous measurements, and one at the time of data collection were used. Taking three consecutive measurements is assumed to improve the reliability of the data.

### 2.8. Conceptual Framework

**Figure 1:**
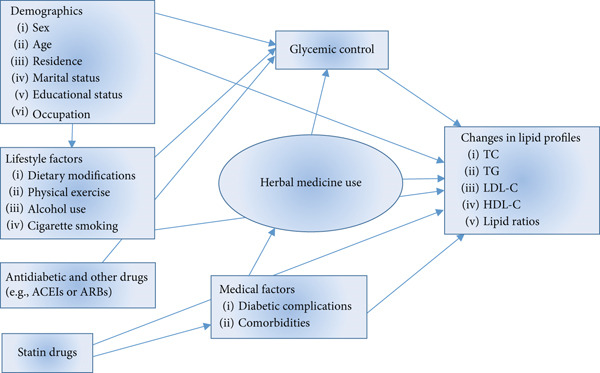
Conceptual framework illustrating the interactions between herbal medicine use and lipid profiles, considering multiple confounding factors.

The interactions between herbal drug use (predictor variable), lipid profile changes (outcome variables), and confounding variables are shown in Figure 1. This conceptual framework was developed based on evidence from literatures that illustrate the relationships among the included variables [25, 27, 37–43].

### 2.9. Operational Definitions

*Lipid profiles*: laboratory test results of blood lipids including TC, LDL‐C, HDL‐C, and TG [44].

*Lipid ratio*: the ratios of TC, TG, or LDL‐C to HDL‐C [40, 45].

*Herbal medicine use*: the use of plant‐based remedies in any form (extracts, teas, powders, decoctions, or unmodified form)—supplemental or alternative to conventional diabetes treatments—intending to control blood glucose levels [46].

*Improved lipid profiles*: decreased total TC, TG, LDL‐C levels, or lipid ratios, and increased HDL‐C value [47].

*Consistent use of herbal medicines*: the use of herbal medicines on a regular basis and at least three times a week.

*Occasional use of herbal medicines*: the use of herbal remedies irregularly and/or less frequently than consistent use.

### 2.10. Data Collection Tool, Process, and Quality Control

A structured data collection tool, adapted from different literatures [25, 27, 40], was used. The tool was organized in sections: sociodemographic characteristics, clinical characteristics, and average laboratory test results for blood lipid levels and fasting blood glucose from the last three consecutive visits. Data were collected through patient medical record review and face‐to‐face interviews. Laboratory test results of lipid levels and fasting blood glucose test results, presence of comorbid conditions, and diabetic complications were extracted from the patients′ medical records. Herbal drug use data and others regarding sociodemographic characteristics, disease duration, adherence to dietary recommendations, engagement in physical exercise, health insurance coverage, alcohol use, and cigarette smoking were collected directly from the participants through face‐to‐face interviews. The plant names were reported in Amharic (local language) and translated to English and botanical names in consultation with a senior researcher on traditional and modern medicine at Armauer Hansen Research Institute, Addis Ababa, Ethiopia. Various quality control measures were taken to ensure data reliability and validity. Initially, all components of the data collection tool, especially variables requiring operational definitions, were explained to the data collectors. The data collectors were also trained on the proper use of the data extraction tool and effective techniques for approaching interviewees to minimize response bias.

A pretest was conducted on a small number of patients and their medical records for any possible ambiguities, inconsistencies, or practical problems in the tool, and some amendments were made. Selection bias was managed by using random selection based on the participants′ appearance at the follow‐up clinic, as described in the sampling procedure. Furthermore, each filled‐out questionnaire was checked for completeness, consistency, potential errors, and eligibility criteria before inclusion in the final analysis.

### 2.11. Data Analysis

The data were analyzed using SPSS Version 25. Cases with missing data on outcome variables were excluded. Descriptive values such as percentages and frequency distributions were computed and presented in tables. Outcome variables (lipid levels, lipid ratios, and AIP) were assessed for normality of distribution using the Shapiro–Wilk test, which indicated that the measures were non‐normally distributed (*p* < 0.05). With this note, we compared the median values of the lipid levels, lipid ratios, and AIP between herbal medicine users and nonusers using the Independent Samples Mann–Whitney *U* test, a nonparametric test. Linear regression analysis was conducted to determine the association between herbal drug use and lipid levels, lipid ratios, and the AIP. Bivariate analysis was first conducted on each potential variable, including herbal drug use itself. Next, a more refined multivariate analysis was conducted to examine the association between herbal drug use and lipid levels, ratios, and AIP, adjusting for more potential confounding variables. For each lipid type, lipid ratio, and AIP, variables with a *p* value less than 0.250 in the binary analysis were included in the multivariate analysis [48]. The cut‐off value for statistical significant association was set at *p* < 0.05, with a 95% confidence interval for the *β*‐coefficient.

### 2.12. Ethical Considerations

This study is part of a broader research project, and the proposal was reviewed and approved by the Institutional Ethical Review Board of the College of Medicine and Health Sciences and Comprehensive Specialized Hospital, University of Gondar for ethical soundness and was found ethically acceptable. The ethical clearance letter was awarded by the Research, Technology Transfer, and Community Service Chief Directorate Office of the college on behalf of the ethical review board (Ref. R/T/T/C/Eng./365/12/2023). Besides, a formal request was sent from the chief directorate office to the hospital′s clinical director office, and permission to access the patient data was granted. Additionally, the participants were included only after written informed consent for participation was obtained. Furthermore, no personal identifiers were collected, and all data records were handled confidentially and used solely for the study.

## 3. Results

The number of participants determined in the sample size calculation (416 participants) was approached and recruited, and all of them were eligible based on our eligibility criteria. Some participants were later excluded from the study due to incomplete data in their medical records, and 381 patients were included in the final analysis, with a response rate of 91.6%. Excluded participants did not differ significantly from included participants in age, sex composition, disease duration, and other pertinent baseline data (*p* > 0.05), suggesting that exclusion did not introduce selection bias.

### 3.1. Sociodemographic Characteristics

The mean age of the participants was 60.9 years (SD = 10.8). Males and females were comparable in proportion (185 (48.6%) vs. 196 (51.4%)), and most of the participants, 326 (85.6%), were urban dwellers. In educational status, the majority of the participants, 127 (33.3%), were illiterate, whereas the largest proportion, 132 (34.6%), were housewives in occupation (Table 1).

**Table 1:** Sociodemographic characteristics of the participants.

**Variable**	**Frequency**	**Percentage**
Age, mean (SD), year	60.9 (10.8)	—
Sex		
Male	185	48.6
Female	196	51.4
Residence		
Urban	326	85.6
Rural	55	14.4
Marital status		
Married	323	84.8
Unmarried	58	15.2
Educational level		
Illiterate	127	33.3
Primary education	91	23.9
Secondary education	75	19.7
Tertiary education	88	23.1
Occupation		
Farmer	34	8.9
Housewife	132	34.6
Government employee	83	21.8
Private employee	55	14.4
Trader	46	12.1
Other	31	8.1

Abbreviation: SD, standard deviation.

### 3.2. Clinical Characteristics

About 253 (66.4%) of the participants followed diet recommendations, while 171 (44.9%) of the subjects reported engagement in regular physical exercise. Furthermore, almost all of the patients were adherent to their clinical follow‐up appointments, and 298 (78.2%) reported having health insurance coverage. Only 43 (11.3%) and 18 (4.7%) of the participants reported alcohol use and cigarette smoking, respectively. Of the total, 141 (37.0%) of respondents reported that they had used herbal medications since their diagnosis, and the majority of them were active users during the study (127 of 141). The majority of herbal drug users reported that they use the herbal remedies concomitant with conventional treatments. The mean duration since the patients were diagnosed with DM was 7.63 years (SD = 4.33). About 263 (69.0%) of the participants had comorbid illnesses, while 143 (37.5%) of them had diabetic complications. The patients were on different antidiabetic drug regimens. The highest proportion of patients, 121 (31.8%), were on the combination of metformin and glibenclamide, followed by those on metformin and NPH insulin (21.0%). Besides, 177 (46.5%) and 225 (59.1%) patients were on ACEI or ARB and statin drugs, respectively. The mean FBG level of participants was 136.67 mg/dL (SD = 29.24). The medians (IQR) of TC, TG, LDL‐C, and HDL‐C were 181.33 (39.63), 148.91 (40.50), 105.05 (34.50), and 46.12 (13.82) mg/dL, respectively. The medians (IQR) of TC/HDL‐C, TG/HDL‐C, and LDL‐C/HDL‐C ratios were 3.95 (1.34), 3.27 (1.41), and 2.22 (1.12), respectively. Regarding the AIP, the median (IQR) value was 0.51 (0.19) (Table 2).

**Table 2:** Clinical characteristics of the participants.

**Variables**	**Frequency**	**Percentage**
Follow diet recommendations		
Yes	253	66.4
No	128	33.6
Physical exercise		
Yes	171	44.9
No	210	55.1
Adhere to clinical follow up		
Yes	357	93.7
No	24	6.3
Health insurance coverage		
Yes	298	78.2
No	83	21.8
Alcohol use		
Yes	43	11.3
No	338	88.7
Cigarette smoking		
Yes	18	4.7
No	363	95.3
Herbal drug use since diagnosis		
Yes	141	37.0
No	240	63.0
Disclosed the herbal medicine use to your physician (*n* = 141)		
Yes	35	24.8
No	106	75.2
Duration of herbal drug use, mean (SD), year		
	2.56 (1.75)	—
Herbal drug use currently (*n* = 141)		
Yes	127	90.1
No	14	9.9
Use of herbal medicine (*n* = 141)		
Concomitantly	132	93.6
Alternatively	9	6.4
Frequency of herbal medication use alongside conventional treatment (*n* = 127)		
Consistently	85	66.9
Occasionally	42	33.1
Time since DM diagnosed, mean (SD), year		
	7.63 (4.33)	—
Comorbid conditions		
Yes	263	69.0
No	118	31.0
Diabetic complications		
Yes	143	37.5
No	238	62.5
ACEI or ARB drug		
Yes	177	46.5
No	204	53.5
Statin drug use		
Yes	225	59.1
No	156	40.9
Antidiabetic medication regimen		
Metformin	86	22.6
Metformin and glibenclamide	121	31.8
Metformin and NPH insulin	80	21.0
NPH insulin	79	20.7
Others	15	3.9
FBG level, mean (SD), mg/dL	136.67 (29.24)	—
TC, median (IDQ), mg/dL	181.33 (39.63)	—
TG, median (IQR), mg/dL	148.91 (40.50)	—
LDL‐C, median (IQR), mg/dL	105.05(34.50)	—
HDL‐C, median (IQR), mg/dL	46.12 (13.82)	—
TC/HDL‐C ratio, median (IQR)	3.95 (1.34)	—
TG/HDL‐C ratio, median (IQR)	3.27 (1.41)	—
LDL‐C/HDL‐C ratio, median (IQR)	2.22 (1.12)	—
Atherogenic index of plasma (AIP)	0.51 (0.19)	—

Abbreviations: ACEI, angiotensin converting enzyme inhibitor; ARB, angiotensin receptor blocker; DM, diabetes mellitus; FBG, fasting blood glucose; HDL‐C, high‐density lipoprotein cholesterol; IQR, interquartile range; LDL‐C, low‐density lipoprotein cholesterol; SD, standard deviation; TC, total cholesterol; TG, triglycerides.

### 3.3. Medicinal Plants Reported by the Participants

The patient‐reported data showed that Type 2 diabetic patients use various medicinal plants in different preparations, with *Trigonella foenum-graecum* being the most commonly reported plant. *Moringa stenopetala* and *Zingiber officinale* were the second most frequently reported medicinal plants to be used by the respondents (each reported by 24 respondents), followed by *Cinnamomum cassia*, *Thymus schimperi*, and *Mangifera indica*. Other additional medicinal plants were also reported by a smaller number of patients to be used as an integral part of the conventional treatments (Table 3).

**Table 3:** Medicinal plants that were reported to be used by Type 2 diabetic patients and ways of use.

**English (Amharic) name**	**Binomial name with family**	**Number of patients**	**Way of use**
Fenugreek (abish)	*Trigonella foenum-graecum* L. (Fabaceae)	47	• Seeds are soaked in water for a night, and the water is taken on an empty stomach. • The powder of dried seeds is mixed with food, honey, or porridge. • The seeds are boiled and used as tea.
Moringa (shiferaw)	*Moringa stenopetala* (Bak. f.) Cufod. (Moringaceae)	24	• The dried leaf powder is mixed with water or food. • Fresh leaves are boiled and used as tea.
Ginger (zingibil)	*Zingiber officinale* Roscoe *(*Zingiberaceae)	24	• Fresh or dried and powdered ginger is boiled and used as tea.
Cinnamon (kerefa)	*Cinnamomum cassia* Presl (Lauraceae)	15	• Intact or powdered bark of the plant are consumed as tea.
Thyme (tosign)	*Thymus schimperi* Ronniger (Lamiaceae)	12	• Leaves are served as tea or mixed with honey and used.
Mango leaves (mango kitel)	*Mangifera indica* L. (Anacardiaceae)	9	• Fresh mango leaves are soaked in water for a night and the water is drunk. • Dried and powdered leaves are taken with warm water.
	*Rumex abyssnicus* Jacq. (Poligonaceae)	7	• The leaves or root parts are boiled and taken as tea.
Garden cress (feto)	*Lepidium sativum* Linn*. (Cruciferae)*	6	• Seeds are soaked in water and taken. • Powdered seeds are taken with honey.
Clove (kirnfud)	*Syzygium aromaticum*. (L.) Merr. and L.M. Perry (Myrtaceae)	6	• Buds are boiled and used as tea.
Pineapple leaf (ananas kitel)	*Ananas comosus* (L.) Merr. (Bromeliaceae)	5	• The leaves are boiled in water, and the water is taken.
Flaxseed or Linseed (telba)	*Linum usitatissimum* L*. (*Linaceae)	4	• Seeds are powdered and added to water or porridge.
Rosemary (rosemary)	*Salvia rosmarinus* Spenn. (Lamiaceae)	4	• Fresh leaves are boiled in water and used as tea. • The leaves are added as a spice in food.
Rue (tena adam)	*Ruta chalepensis* L. (Rutaceae)	3	• Leaves are boiled and consume as tea. • Leaves are added to coffee and drunk.
Red teff porridge (key tef)	*Eragrostis tef* (Zuccagni) Trotter (Poaceae)	2	• The flour is used as porridge.
Pumpkin seeds (yeduba fire)	*Cucurbita pepo* L. (Cucurbitaceae)	2	• Roasted or raw seeds are eaten.
African basil (damakase)	*Ocimum lamiifolium* Hochst. ex Benth. (Lamiaceae)	2	• Fresh leaves are boiled and used as tea.
Turmeric (erd)	*Curcuma longa* L. (Zingiberaceae)	1	• The rhizome is powdered, mixed with honey, and consumed. • The powder is added to food as a spice.

### 3.4. Comparison of Lipid Levels and Rations Between Herbal Medicine Users and Nonusers

The median TC level of herbal medicine users was significantly lower than that of nonusers (173.39 mg/dL vs. 187.29 mg/dL, *p* < 0.01). The median TG value was also significantly lower in herbal medicine users compared with nonusers (146.36 mg/dL vs. 154.92 mg/dL, *p* < 0.01). Similarly, the median LDL‐C level of herbal medicine users was considerably lower than that of nonusers (96.86 mg/dL vs. 105.50 mg/dL, *p* < 0.01). The median ratios of herbal drug users and nonusers were also compared. Accordingly, the median TC/HDL‐C ratio of participants who reported using herbal medicine was significantly lower than that of those who did not repot (3.64 vs. 4.07, *p* < 0.05). Likewise, the median TG/HDL‐C and LDL‐C/HDL‐C ratios were significantly lower among herbal medicine users compared with nonusers (*p* < 0.01). Besides, the median AIP was also significantly reduced in herbal medicine users (0.48 vs. 0.54, *p* < 0.01) (Table 4).

**Table 4:** Results of the independent samples Mann–Whitney *U* test showing differences in median lipid levels and lipid ratios between herbal medicine users and nonusers.

**Lipids and lipid rations**	**Herbal medicine users**		**Herbal medicine nonusers**		**p** **value**
	**Median (IQR)**	**Mean rank**	**Median (IQR)**	**Mean rank**	
Total cholesterol (mg/dL)	173.59 (43.00)	170.80	187.26 (39.22)	202.87	0.006
Triglycerides (mg/dL)	146.36 (39.07)	170.24	154.92 (43.89)	203.20	0.005
Low‐density lipoprotein (mg/dL)	96.86 (28.50)	167.08	105.50 (35.41)	205.05	0.001
High‐density lipoprotein (mg/dL)	46.27 (13.50)	205.39	44.79 (13.95)	182.55	0.039
TC/HDL‐C	3.64 (1.29)	167.55	4.07 (1.39)	204.78	0.001
TG/HDL‐C	3.05 (1.36)	169.26	3.49 (1.46)	203.77	0.003
LDL‐C/HDL‐C	2.05 (0.91)	169.10	2.32 (1.15)	203.87	0.003
Atherogenic index of plasma (AIP)	0.48 (0.19)	169.09	0.54 (0.18)	203.87	0.003

Abbreviations: LDL‐C/HDL‐C, low‐density lipoprotein cholesterol to high‐density lipoprotein cholesterol ratio; TC/HDL‐C, total cholesterol to high‐density lipoprotein cholesterol ratio; TG/HDL‐C, triglycerides to high‐density lipoprotein cholesterol ratio.

### 3.5. Linear Regression Analyses

#### 3.5.1. Bivariate Analysis of Confounding Variables

The analysis demonstrated that several factors were significantly associated with TC, TG, LDL‐C, or HDL‐C levels and TC/HDL‐C, TG/HDL‐C, and LDL‐C/HDL‐C ratios. Engagement in physical exercise and statin drug use were negatively associated with TC test results. Conversely, FBG levels, disease duration, and comorbid conditions were positively associated with TC levels. Following diet recommendations and physical exercise were negatively associated with TG levels, whereas comorbid conditions, diabetic complications, and FBG were positively associated. Physical exercise and statin drug use were negatively associated with LDL‐C levels, whereas age, disease duration, and FBG were positively associated. Dietary recommendations and physical exercise were positively correlated with HDL‐C results, whereas FBG showed a negative association. Dietary modifications, physical exercise, and statin drug use were negatively associated with TC/HDL‐C ratio, and conversely, age, disease duration, comorbidity, diabetic complications, and FBG were positively associated. Following diet recommendations, physical exercise, and statin drug use were associated with lower TG/HDL‐C ratios, whereas being married, comorbid diseases, diabetic complications, and FBG were associated with higher ratios. Regarding LDL‐C/HDL‐C ratio, positive associations were observed with male age, comorbidities, and FBG, and a negative association with sex (female). On the other hand, dietary modifications, physical exercise, and statin drug use were negatively associated with LDL‐C/HDL‐C ratios. The bivariate regression analysis also demonstrated that marital status, dietary recommendations, physical exercise, and statin drug use were significantly negatively associated with the AIP. In contrast, diabetic complications and fasting blood glucose levels were significantly positively associated with the index value (Table 5).

**Table 5:** Bivariate regression analysis of confounding factors.

**Variables**	**TC,** **β** **(95% CI)**	**TG,** **β** **(95% CI)**	**LDL-C, *β* (95% CI)**	**HDL-C,** **β** **(95% CI)**	**TC/HDL-C,** **β** **(95% CI)**	**TG/HDL-C,** **β** **(95% CI)**	**LDL-C/HDL-C,** **β** **(95% CI)**	**AIP,** **β** **(95% CI)**
Sex	1.82 (−4.94, 8.57)	−0.25 (−6.51, 6.01)	−2.61 (−8.50, 3.28)	1.95 (−0.11, 4.01)	−0.21 (−0.44, 0.02)	−0.21 (−0.46, −0.04) ^∗^	−0.22 (−0.41, −0.02) ^∗^	0.027 (0.055, 0.001)
Age (year)	0.17 (−0.14, 0.49)	0.12 (−0.17, 0.41)	0.28 (0.01, 0.56) ^∗^	−0.08 (−0.18, 0.02)	0.01 (0.00, 0.02^)^^∗^	0.01 (−0.002, 0.02)	0.01 (0.0001, 0.02) ^∗^	0.001 (0.000, 0.002)
Residence	3.68 (−5.94, 13.29)	−2.73 (−6.17, 11.63)	3.24 (−5.14, 11.62)	−0.57 (−3.51, 2.37)	0.22 (−0.12, 0.55)	0.14 (−0.22, 0.50)	0.18 (−1.00, 0.46)	−0.014 (−0.055, 0.026)
Marital status	−8.79 (−18.16, 0.58)	−6.95 (−1.74, 15.63)	−8.04 (−18.21, 0.12)	−2.22 (−5.08, 0.65)	0.07 (−0.26, 0.40)	0.40 (0.06, 0.75) ^∗^	0.001 (−0.27, 27)	−0.044 (−0.082, −0.005) ^∗^
Educational status	−1.00 (−3.91, 1.91)	−1.52 (−4.21, 1.17)	−0.76 (−3.29, 1.78)	0.27 (−0.62, 1.16)	−0.03 (−0.13, 0.08)	−0.04 (−0.15, 0.07)	−0.002 (−0.09, 0.08)	−0.006 (−0.018, 0.006)
Occupation	−0.89 (−3.25, 1.46)	−1.95 (−4.12, 0.23)	0.08 (−1.97, 2.14)	−0.35 (−1.07, 0.37)	0.02 (−0.06, 0.11)	−0.01 (−0.09, 0.08)	0.03 (−0.04, 0.10)	−0.001 (−0.010, 0.009)
Disease duration (year)	0.80 (0.03, 1.58) ^∗^	0.29 (−0.43, 1.01)	0.85 (0.18, 1.53) ^∗^	−0.13 (−0.37, 0.11)	0.03 (0.002, 0.06) ^∗^	0.02 (−0.01, 0.05)	0.02 (0.000, 0.05)	−0.002 (−0.001, 0.005)
Diet recommendations	−4.44 (−11.58, 2.71)	−6.67 (−13.26, −0.08) ^∗^	−5.02 (−11.24, 1.20)	2.20 (0.03, 4.38) ^∗^	−0.31 (−0.56, −0.06) ^∗^	−0.30 (−0.56, −0.04) ^∗^	−0.23 (−0.44, −0.02) ^∗^	−0.040 (−0.069, −0.010) ^∗∗^
Physical exercise	−8.42 (−15.17, −1.68) ^∗^	−7.65 (−13.90, −1.41) ^∗^	−9.05 (−14.91, −3.20) ^∗∗^	2.58 (0.52, −4.64) ^∗^	−0.44 (−0.67, −0.21) ^∗∗∗^	−0.37 (−0.62, −0.12) ^∗∗^	−0.37 (−0.56, −0.18) ^∗∗∗^	−0.046 (−0.074, −0.018) ^∗∗^
Health insurance coverage	−2.30 (−10.49, 5.89)	2.75 (−4.83, 10.32)	−1.63 (−8.77, 5.51)	−1.00 (−3.50, 1.50)	0.02 (−0.27, 0.30)	0.15 (−0.15, 0.45)	−0.04 (−0.28, 0.19)	0.019 (−0.015, 0.053)
Alcohol use	0.30 (−10.39, 10.99)	−2.61 (−12.49, 7.28)	1.81 (−7.51, 11.12)	−1.05 (−4.31, 2.21)	0.13 (−0.24, 0.51)	0.03 (−0.37, 0.42)	0.11 (−0.20, 0.42)	0.005 (−0.039, 0.050)
Comorbid conditions	7.23 (−0.06, 14.53) ^∗^	6.98 (0.24, 13.73) ^∗^	1.81 (−7.51, 11.12)	−0.54 (−2.78, 1.70)	0.26 (0.01, 0.52) ^∗^	0.27 (0.000, 0.54) ^∗^	0.22 (0.01, 0.44) ^∗^	0.026 (0.004, 0.057)
Diabetic complications	3.55 (−3.43,10.52)	7.94 (1.53, 14.3.6) ^∗^	4.17 (−1.91, 10.24)	−1.73 (−3.86, 0.39)	0.25 (0.01, 0.49) ^∗^	0.33 (0.07, 0.59) ^∗^	0.18 (−0.02, 0.38)	0.041 (0.012, 0.070) ^∗∗^
Average FBG level	0.29 (0.18, 0.40) ^∗∗∗^	0.41 (0.31, 0.51) ^∗∗∗^	0.27 (0.17, 0.36) ^∗∗∗^	−0.10 (−0.13, −0.06) ^∗∗∗^	0.02 (0.01, 0.02) ^∗∗∗^	0.019 (0.015, 0.023) ^∗∗∗^	0.01 (0.01, 0.02) ^∗∗∗^	0.001 (0.002, 0.003) ^∗∗∗^
ACEI or ARB drug use	−4.02 (−10.79, 2.75)	−3.00 (−9.27, 3.27)	−0.79 (−6.69, 5.12)	−0.67 (−2.74, 1.40)	0.10 (−0.14, 0.34)	0.14 (−0.11, 0.39)	0.09 (−0.11, 0.29)	0.001 (−0.028, 0.029)
Statin drug use	−9.22 (−16.03, −2.40) ^∗∗^	−5.35 (−11.69, 1.00)	−10.28 (−16.18, −4.38) ^∗∗^	1.35 (−0.75, 3.45)	−0.39 (−0.63, −0.15) ^∗∗^	−0.25 (−0.51, −0.001) ^∗^	−0.36 (−0.56, −0.17) ^∗∗∗^	−0.029 (−0.057, −0.001) ^∗^
Antidiabetic drug regimens	−0.26 (−3.16, 2.65)	−1.23 (−3.92, 1.46)	−1.52 (−4.05, 1.01)	0.25 (−0.64, 1.14)	−0.06 (−0.16, 0.05)	−0.08 (−0.19, 0.03)	−0.07 (−0.16, 0.01)	−0.007 (−0.019, 0.005)
Duration of herbal medicine drug	0.54 (−2.97, 4.05)	−0.46 (−3.27, 2.34)	1.63 (−1.50, 4.75	−0.80 (−1.78, 0.18)	0.06 (−0.05, 0.17	0.04 (−0.07, 0.14	0.06 (−0.03, 0.15)	0.009 (−0.003, 0.022)
Frequency of herbal drug use	1.02 (−11.97, 14.02)	−0.39 (−10.78, 9.99)	−2.10 (−13.71, 9.51)	0.80 (−2.86, 4.46)	−0.11 (−0.51, 0.30)	−0.04 (−0.43, 0.36)	−0.12 (−0.44, 0.19)	−0.006 (−0.055, 0.043)

Abbreviations: ACEI, angiotensin converting enzyme inhibitor; AIP, atherogenic index of plasma; ARB, angiotensin receptor blocker; FBG, fasting blood glucose; HDL‐C, high‐density lipoprotein cholesterol; LDL‐C, low‐density lipoprotein cholesterol; TC, total cholesterol; TG, triglycerides; TC/HDL‐C, total cholesterol to high‐density lipoprotein cholesterol ratio; TG/HDL‐C, triglycerides to high‐density lipoprotein cholesterol ratio.

^∗^ *p* < 0.05.

^∗∗^ *p* < 0.01.

^∗∗∗^ *p* < 0.001.

#### 3.5.2. Bivariate and Multivariate Analysis of the Effects of Herbal Drug Use on Lipid Levels and Ratios

The bivariate and multivariate analyses showed that herbal medicine use was significantly associated with reductions in TC, TG, and LDL‐C levels as well as TC/HDL‐C, TG/HDL‐C, and LDL‐C/HDL‐C ratios. The bivariate analysis showed that herbal medicine use was associated with a 7.78 mg/dL decrease in TC level (*β* = −7.78, *p* < 0.05). The analysis also revealed that using herbal drugs was correlated with a 9.12 mg/dL (*β* = −9.12, *p* < 0.01) and a 6.78 mg/dL (*β* = −6.78, *p* < 0.05) reductions in TG and LDL‐C levels, respectively, and with a 1.68 mg/dL (*β* = 1.68, *p* < 0.05) increase in HDL‐C value. Similarly, the bivariate analysis showed that using herbal drugs was associated with a 0.37 reduction in TC/HDL‐C and TG/HDL‐C ratios (*β* = −0.37, *p* < 0.01) and a 0.29 reduction in LDL‐C/HDL‐C ratio (*β* = −0.29, *p* < 0.01). Likewise, the multivariate analysis demonstrated that TC levels were decreased by 6.84 mg/dL in the patients who used herbal medicine compared with nonusers (*β* = −6.84, *p* < 0.05). TG values were also reduced by 8.69 mg/dL (*β* = −8.69, *p* < 0.01) and LDL‐C levels by 6.75 mg/dL (*β* = −6.75, *p* < 0.05) in herbal medicine users, whereas HDL‐C values were increased by 1.59 mg/dL (*β* = 1.59, *p* < 0.05). Additionally, the TC/HDL‐C, TG/HDL‐C, and LDL‐C/HDL‐C ratios were lower among herbal medicine users by 0.32 (*β* = −0.32, *p* < 0.05), 0.34 (*β* = −0.34, *p* < 0.01), and 0.23 (*β* = −0.23, *p* < 0.05), respectively, compared with nonusers. Moreover, both the bivariate and multivariate linear regression models showed that participants who used herbal medicines had significantly lower AIP values. In the bivariate model, AIP values of herbal medicine users were 0.043 lower, on average, than those of nonusers (*β* = −0.043, *p* < 0.01). Similarly, the multivariate model showed that the values were decreased by 0.041 in herbal medicine users (*β* = −0.041, *p* < 0.01), compared with those of nonusers (Table 6).

**Table 6:** Bivariate and multivariate linear regression analysis of the effect of herbal medicine use on lipid parameters in Type 2 diabetic patients

**Analysis**	**TC,** **β** **(95% CI)**	**TG,** **β** **(95% CI)**	**LDL-C,** **β** **(95% CI)**	**HDL-C,** **β** **(95% CI)**	**TC/HDL-C,** **β** **(95% CI)**	**TG/HDL-C,** **β** **(95% CI)**	**LDL-C/HDL-C,** **β** **(95% CI)**	**AIP,** **β** **(95% CI)**
Bivariate	−7.78 (−14.74, −0.82) ^∗^	−9.12 (−15.53, −2.70) ^∗∗^	−6.78 (−12.84, −0.71) ^∗^	1.68 (0.15, 3.51) ^∗^	−0.37 (−0.81, −0.13) ^∗∗^	−0.37 (−0.62, −0.11) ^∗∗^	−0.29 (−0.49, −0.09) ^∗∗^	−0.043 (−0.072, −0.015) ^∗∗^
Multivariate	−6.84 (−13.22, −0.46) ^∗^	−8.69 (−15.62, −2.76) ^∗∗^	−6.75 (−12.56, −0.94) ^∗^	1.59 (0.12, 3.30) ^∗^	−0.32 (−0.55, −0.09) ^∗∗^	−0.34 (−0.58, −0.10) ^∗∗^	−0.23 (−0.44, −0.02) ^∗^	−0.041 (−0.071, −0.011) ^∗∗^

*Note:* The multivariate analysis was adjusted for potential confounding factors. The factors that were found significantly associated in the binary analysis (Table 5) plus other variables with *p* < 0.250 were considered. Accordingly, significantly associated factors in the binary analysis plus for TC: age, marital status, following diet recommendations, and comorbid conditions; TG: marital status, statin drug use, and occupation; LDL‐C: marital status, following diet recommendations, diabetic complications, and comorbid conditions; HDL‐C: sex, age, marital status, statin drug use, and diabetic complications; TC/HDL‐C: sex and residence; TG/HDL‐C: age, disease duration, and antidiabetic medications; LDL‐C/HDL‐C: residence, disease duration, and diabetic complications; and AIP: sex, age, disease duration, comorbid conditions, and diabetic complications were included in the multivariate analyses.

^∗^ *p* < 0.05.

^∗∗^ *p* < 0.01.

## 4. Discussion

Hospital‐based studies in Ethiopian health settings have demonstrated that considerable proportions of T2DM patients on clinical follow‐up also use herbal medicines concomitant to their conventional medications [25–28]. Besides, several medicinal plants claimed to have antidiabetic effects in Ethiopian traditional medicine have been tested for their antidiabetic and antihyperlipidemic effects using animal models [29–31, 49–51]. However, observational or clinical trial studies on their health impacts remain very limited, and evidence in this regard is scarce. Thus, this study aims to evaluate the effects of herbal medicine use on lipid parameters in patients with T2DM.

The nonparametric analysis (Table 4) showed that herbal medicine users had significantly lower median levels of TC, TG, and LDL‐C compared with nonusers, whereas their HDL‐C levels were significantly higher. The analysis also showed that TC/HDL‐C, TG/HDL‐C, and LDL‐C/HDL‐C ratios of herbal medicine users were significantly lower compared with those of the patients who reported not using herbal remedies. These results suggest that herbal medicine use was associated with beneficial effects by decreasing lipids that could increase T2DM‐related complications. Moreover, the median AIP was significantly reduced among herbal medicine users compared with nonusers. Although the median AIP values for both herbal medicine users (0.48) and nonusers (0.54) fall within the high atherogenic risk range (AIP > 0.21) [52], the significant reduction in AIP among herbal users suggests a potential beneficial effect of herbal medicine use on lipid profiles. As shown in Table 6, the linear regression analysis conducted accounting for potential confounding factors also demonstrated that herbal medicine use was significantly negatively associated with TC, TG, and LDL‐C values, whereas positively associated with HDL‐C levels. These favorable associations further suggest that the use of herbal medicines may have beneficial effects in improving lipid profiles among patients with Type 2 diabetes. The regression analysis results also imply that herbal medicine use was significantly correlated with reductions in TC/HDL‐C, TG/HDL‐C, and LDL/HDL‐C ratios. Effects on lipid ratios are more reliable than on individual lipid values as they reflect the impacts on the complex interplay underlying the metabolism of lipids more accurately [53]. The lipid ratios are considered to represent atherogenic to antiatherogenic lipoprotein ratios [45]. Therefore, the reductions in the lipid ratios in herbal medication users imply the beneficial effects of the herbal remedies in optimizing the lipid profiles of T2DM patients. Furthermore, the multivariate model showed that the AIP was significantly lower in herbal medicine users than in nonusers (*β* = −0.041, *p* < 0.01). AIP has been recognized as a novel and reliable indicator of the risk of atherosclerosis and associated cardiovascular diseases [54–57]. In line with this, the present finding suggests that herbal medicine use may contribute to lowering atherogenic risk and offer cardioprotective effects among Type 2 diabetic patients.

*T. foenum-graecum*, *M. stenopetala*, *Z. officinale*, and *C. cassia* were mentioned more frequently for use by the herbal medicine users (Table 3), and in vivo and/or in vitro studies demonstrated that they have antidiabetic, antihyperlipidemic, or antioxidant activities [23, 58–63], which are important for improving lipid profiles [22, 24, 64]. Optimal glycemic control could help improve lipid profiles in T2DM patients [65]. The observed favorable effects on lipid profiles may, therefore, be attributed to the antihyperglycemic properties of the medicinal plants used, supporting previous reports. Moreover, the findings of our study are consistent with reports of previous clinical trials that have evaluated the effects of herbal drug use, including the medicinal plants reported in this study. These studies have reported that herbal drug use is associated with improvements in lipid profiles. Specifically, they have shown reductions in TC, TG, and LDL‐C levels along with increases in HDL‐C levels, effects essentially defined collectively as an improved lipid profile [42, 43]. The results of this study may dictate that the concurrent use of herbal remedies with conventional antidiabetic medications improves lipid profiles while also complementing glycemic control.

Medicinal plants contain a variety of active phytochemicals that can improve lipid profiles. Phytochemicals such as flavonoids, alkaloids, saponins, and polyphenols help lower lipid levels in the body through different mechanisms [33, 34]. Previous studies have highlighted numerous mechanisms through which medicinal plants exert their beneficial effects on blood lipids, which are largely attributed to their phytochemical contents. *T. foenum-graecum* increases biliary excretion of cholesterol and also decreases cholesterol and fat absorption [43]. *Z. officinale* inhibits cholesterol biosynthesis and promotes its metabolic conversion to bile acids, and this results in enhanced excretion of cholesterol with feces [66]. *M. stenopetala* is also proposed to decrease fatty acid synthesis and cholesterogenesis, whereas increasing HDL‐C levels possibly via inhibition of pancreatic lipase and cholesterol esterase [59]. On the other hand, insulin resistance plays a pivotal role in the pathophysiology of Type 2 diabetes and is attributed to diabetic dyslipidemia. The resistance is accompanied by elevation of serum insulin and reduction of beta‐cells and leads to impaired regulation of circulating lipoprotein and glucose levels [67, 68]. Therefore, the observed reduction in lipid levels in the herbal medicine users may be attributed to improved insulin sensitivity and, hence, improved insulin and lipoprotein regulation. Furthermore, the medicinal plants reported to be used by the herbal medicine users have been demonstrated to have antioxidant and anti‐inflammatory activities [60, 69–71]. These activities are known to have useful biological effects in optimizing blood lipid levels [20, 22, 24, 64]. Therefore, the favorable effect of herbal medicine use on lipid profile as observed in this study may be attributed to the diverse biological activities of medicinal plants, which could work in concert to enhance lipid metabolism and hence ultimately cardiovascular health.

Despite their beneficial effects, medicinal plants may also pose untoward or toxic effects. Many medicinal plants mentioned for use by the herbal medicine users in this study have toxic effects, as reported by previous studies [72–81]. Medicinal plants may contain some bioactive compounds implicated in nephrotoxicity, hepatotoxicity, or gastrointestinal disturbances, notably when taken in high amounts or for extended time. Unstandardized dosage and preparation increase the risk of toxicity. Besides, some medicinal plants may interact with prescribed medications, resulting in adverse effects or reduced efficacy [82]. Although assessing side effects was not the primary objective of this study, the low rate of disclosure by participants, combined with the documented toxicities and associated risks of nondisclosure, prompted us to highlight this issue. In this study, most of the herbal medicine users did not disclose to healthcare providers. Only 35 (24.8%) of herbal drug users reported disclosure to their physicians. Nondisclosure of herbal drug use may pose significant risks, as the unchecked use of herbal remedies with conventional medications can result in harmful drug–herb interactions, potentially leading to toxicity or reduced therapeutic efficacy of the conventional drugs [83]. Given that only 24.8% of herbal medicine users disclosed to their physicians, we recommend implementing specific strategies to improve disclosure. These may include training healthcare providers to ask about herbal medicine use during routine follow‐ups using a structured checklist and creating a supportive environment that encourages open communication between patients and providers.

This study has a significant strength and some limitations. Previous studies conducted in Ethiopian health settings primarily focused on the prevalence of herbal drug use and associated factors in T2DM patients. However, evidence on any beneficial effects of herbal medicine under actual use circumstances is limited. This is, therefore, the first of its kind to assess the impacts of herbal drug use, specifically on lipid profiles, in the real herbal medicine use practice among T2DM patients, and it is expected to provide valuable insight. This is considered a strength of the study as it contributes to addressing the limited evidence on the health effects of herbal medicine use under uncontrolled and practical conditions among diabetic patients. However, the study also has limitations. The results of this study may be affected by a potential source of bias. Patients may answer in favor of some variables, such as adherence to dietary recommendations and engagement in physical exercise, and this may affect the analysis results because these factors were considered potential confounders. Additionally, patients may lack the confidence to disclose their use of herbal medicines, which could lead to an underestimation of the proportion of herbal medicine users and affect the final analysis of the impacts on lipid levels, lipid ratios, and AIP. This study is a cross‐sectional study conducted at only one hospital. Therefore, this does not show the nature of the correlation between herbal medicine use and lipid profiles, and the results obtained should not be fully inferred to patients with Type 2 diabetes in general [48]. Therefore, we recommend conducting further multicenter cross‐sectional and controlled trial studies in Ethiopian health settings for more comprehensive and conclusive results.

## 5. Conclusion

In conclusion, the use of herbal medicines was associated with significant reductions in TC, TG, and LDL‐C levels, as well as in the TC/HDL‐C, TG/HDL‐C, and LDL‐C/HDL‐C ratios and AIP, while also associated with a significant increase in HDL‐C level. The results imply that herbal remedies may have beneficial effects in optimizing serum lipid levels in T2DM patients and could ultimately help reduce associated cardiovascular risks. The study confirmed the beneficial effects of herbal medicine use in real‐world patient practice, where evidence remains limited in our country. However, because this study was cross‐sectional and carried out at a single site, we recommend conducting more rigorous, multicenter observational and trial studies in the Ethiopian healthcare settings to generate more comprehensive and conclusive results. Additionally, we recommend that policymakers and healthcare providers promote the integration of evidence‐based herbal medicine use into diabetes care. We also suggest strategies such as training healthcare providers to ask about herbal medicine use during routine follow‐ups using a structured checklist and creating a supportive environment for patient–provider communication to improve herbal medicine use disclosure.

## Disclosure

All authors approved the manuscript for publication and agreed to be accountable for its content and conclusions.

## Conflicts of Interest

The authors declare no conflicts of interest.

## Author Contributions

A.B.A., T.M.B., T.J.A., M.F.M., and G.L.L. contributed to the conception, design, and development of the study proposal. A.T.B., H.S.A., K.G.T., Y.Z.M., E.A.A., H.T.A., and Y.G.L. followed the data collection process and conducted regular quality checks. A.B.A. performed the data analysis and interpretation of the findings. All authors contributed to drafting the initial report. A.B.A. wrote the manuscript, and then, A.T.B., H.S.A., and G.L.L. reviewed it before submission and provided feedback for which the revisions were made.

## Funding

No funding was received for this manuscript.

## Data Availability

The data that support the findings of this study are available from the corresponding author upon reasonable request.
